# Type 1 Diabetes/Hidradenitis Suppurativa Comorbidity—A Population-Based Study

**DOI:** 10.3390/jcm14082625

**Published:** 2025-04-11

**Authors:** Shany Sherman, Ron Slama, Danielle Bar, Yochai Schonmann, Arnon D. Cohen, Yossef H. Taieb, Daniel Mimouni, Alon Peretz, Hadar Duskin-Bitan

**Affiliations:** 1 Division of Dermatology, Rabin Medical Center, Beilinson Hospital, Petach Tikva 4941492, Israel; yossi.taieb.260287@gmail.com (Y.H.T.); mimouni@post.tau.ac.il (D.M.); 2 Faculty of Medicine, Tel Aviv University, Tel Aviv 6997801, Israel; ronslama@mail.tau.ac.il (R.S.); daniellebar1@mail.tau.ac.il (D.B.); hadarda@clalit.org.il (H.D.-B.); 3 Department of Family Medicine, Faculty of Medicine & Health Sciences, Tel Aviv University, Tel Aviv 6997801, Israel; yochais@clalit.org.il; 4 Department of Quality Measurements and Research, Clalit Health Services, Tel Aviv 6209804, Israel; ardcohen@gmail.com; 5 Faculty of Health Sciences, Ben Gurion University of the Negev, Beer Sheva 8410501, Israel; 6 Division of Community Medical Services, Clalit Health Services, Tel Aviv 6209804, Israel; shalonpe@clalit.org.il; 7 Institute of Endocrinology, Rabin Medical Center, Beilinson Hospital, Petach Tikva 4941492, Israel

**Keywords:** population-based study, nested case control, hidradenitis suppurativa (HS), type 1 diabetes (T1D), odds ratio

## Abstract

**Background**: Type 1 diabetes (T1D) and hidradenitis suppurativa (HS) share several metabolic and inflammatory dysfunctions. Prior studies of the potential link between the diseases either lacked validated T1D diagnoses or established only an indirect association. This study sought to determine the odds of HS developing in patients with a validated diagnosis of T1D and to characterize the clinical features of HS/T1D comorbidity. **Methods**: A population-based nested case-control study was conducted including patients with HS and controls matched 5:1 for age, sex, and primary care clinic. T1D was diagnosed using a specialized algorithm, achieving 90% accuracy. Diagnostic validity was confirmed by diabetes specialists who manually reviewed a random subset of the files. Unadjusted and adjusted odds ratios (OR/aOR) were calculated to determine the odds of incident HS in patients with T1D. **Results**: The study included 10,919 patients with HS and 53,314 controls. A history of T1D was associated with an elevated odds of new-onset HS (OR 1.80 95% CI (1.30–2.40), *p* < 0.001), even after adjusting for demographics and metabolic and autoimmune comorbidities (aORs > 1.7, *p* < 0.001). Patients with HS/T1D comorbidity had higher proportions of autoimmune conditions than patients with HS alone (*p* < 0.001) and a higher mean Charlson Comorbidity Index score than both patients with HS alone (3.5 vs. 0.9, *p* < 0.001) and T1D alone (3.5 vs. 2.2, *p* = 0.004). **Conclusions**: T1D is associated with higher odds of the subsequent development of HS. Awareness of HS/T1D comorbidity is recommended owing to the elevated burden of metabolic and autoimmune conditions.

## 1. Introduction

Hidradenitis suppurativa (HS) is a chronic, inflammatory, debilitating skin disease that typically manifests in young adults [1]. It presents as painful, deep-seated, inflammatory lesions in the apocrine-gland-bearing areas of the body, most commonly the axillary, inguinal, and anogenital regions [2]. The prevalence of HS is unknown but is estimated to range from 0.3% to 1.7%, depending on the study design [1]. The diagnosis is made clinically based on three criteria: characteristic lesions, predilection for flexural sites, and lesion recurrence. In a subset of patients, HS will progress to the formation of scarring, tunnels, and fistulas [1]. The diagnosis is often delayed for years [3], which may further exacerbate the dramatic decrease in quality of life [4].

Recent research described the role of the immune system in the pathogenesis of HS [5]. Levels of pro-inflammatory cytokines, such as tumor necrosis factor alpha (TNF-α), interleukin (IL)-1β, and IL-17, are elevated in lesional tissues [6].

Patients with HS have a high comorbidity burden. HS is associated with metabolic syndrome and dyslipidemia, hypertension, and obesity. A higher prevalence of type 2 diabetes mellitus (T2D) was reported in a group of patients with HS compared to the general population [7]. HS has also been linked to inflammatory bowel disease, spondyloarthropathy, and thyroid disorders [7,8].

Type 1 diabetes mellitus (T1D) is a chronic autoimmune disease that results from insulin deficiency due to destruction of the pancreatic islet beta cells [9]. T1D is one of the most common endocrine and metabolic conditions occurring in childhood. The estimated worldwide prevalence is 0.095% of the general population [10].

Akin to HS, T1D is associated with immune-mediated diseases, such as celiac disease, thyroid disease, psoriasis, and vitiligo [11]. In prior research, the criteria for diagnosing diabetes often varied by the data source, such as physician claims or hospital discharge records [12]. The lack of validation of ICD-10 codes with subsequent misclassification of T1D and T2D can lead to significant bias [13,14,15,16,17,18], particularly among older individuals and patients with atypical presentations, such as obesity-associated T1D. To date, two population-based studies have suggested an association of T1D with HS [19,20]. The first, from Korea, utilized a cross-sectional design, which precluded analysis of temporality and lacked consideration of confounders [19]. The second study identified HS indirectly, by the appearance of cutaneous abscesses, which is not a sine qua non for diagnosis of HS [20]. In both, T1D was diagnosed by unvalidated ICD-10 codes.

The objectives of the present study were to investigate whether patients with T1D are at increased odds of the development of incident HS and to describe the clinical features of HS/T1D comorbidity.

## 2. Materials and Methods

This population-based study was conducted to estimate the likelihood of incident HS developing in patients with T1D. As HS is considered a relatively rare disease (<10% of population), a nested case-control design was chosen in order to detect enough cases (outcome). With this design, the odds ratio (OR) is a good approximation of the hazard ratio (HR) [18]. Data were sourced from Clalit Health Services (CHS), the largest healthcare organization in Israel, with 4,873,374 enrollees as of December 2023. The real-time computerized CHS dataset encompasses comprehensive medical, pharmaceutical, and administrative records. Every encounter within the healthcare system is documented in the patient’s medical record. The database of CHS undergoes continuous updates to ensure a thorough longitudinal perspective on the health of its enrollees, enhancing its value for epidemiological research by minimizing selection and information biases.

Dermatology consultations within CHS are available in three settings: during hospitalization, in outpatient clinics affiliated with hospitals, or in community-based primary care clinics, where independent board-certified dermatologists provide services. Visits to family physicians and pediatricians are free, and community dermatology consultations require only a small quarterly co-payment (~10 USD) with no need for a referral. This streamlined, affordable access contributes to the high rate of dermatologist visits in Israel.

The study protocol was reviewed and approved by the CHS Institutional Review Board (approval number COM2-0212-17).

For the present study, all new cases of HS diagnosed in 2000–2023 were identified. Cases were included when the HS-specific code (ICD-9 705.83) was registered by a CHS dermatologist or, for patients hospitalized in a dermatology ward, when the diagnosis of HS was documented in a discharge letter. The validity of ICD coding in HS has been demonstrated previously [17,18].

The diagnosis of T1D was based on an algorithm designed at the CHS Chronic Disease Center. The algorithm produced a hierarchy of four sub-algorithms. If at least one of the sub-algorithms was positive, the patient was considered to have T1D.

The diagnostic criteria included the following: (1) diagnosis of diabetes before age 18 years and at least 2 purchases of rapid insulin packs within 6 months of diabetes diagnosis, or (2) a positive anti-glutamic acid decarboxylase (GAD) or islet cell antibody test and at least 2 purchases of rapid insulin packs within 6 months of diabetes diagnosis, or (3) a T1D code in the medical records and at least 2 purchases of rapid insulin packs within 6 months of diabetes diagnosis. (4) Patients diagnosed with diabetes between the ages of 18 and 25 years were included if they purchased at least 2 packs of rapid insulin per year for 5 consecutive years.

The validation process was conducted by 2 board-certified endocrinologists and involved the manual review of 50 randomly selected cases for each of the 4 diagnostic criteria, totaling 200 cases out of 13,167 patients with T1D. The accuracy rate for the first three criteria exceeded 95%. The fourth criterion, which was rarely used in the registry, had an accuracy rate of 76%. Overall, the agreement between the endocrinologists and the registry exceeded 90%.

The index time was the year of diagnosis of HS. In that year, for each study patient, five control subjects without a diagnosis of HS matched for age, sex, and primary care clinic were selected. Matching for primary care clinic was important to account for unmeasured confounders, such as the availability of healthcare services, as well as composite variables, such as socioeconomic status and ethnicity.

The characteristics of the study participants were compared across treatment groups. The chi-square test was used for categorical variables when all expected cell frequencies were ≥5; otherwise, Fisher’s exact test was used. Continuous variables were assessed for normality using the Shapiro–Wilk test and visual inspection of histograms and Q-Q plots. If normality was confirmed, Welch’s *t*-test was used to compare groups, accounting for unequal variances. For non-normally distributed variables, the Mann–Whitney U test was applied. The association between HS and T1D was assessed by conditional logistic regression with calculation of odd ratios (ORs) and 95% confidence intervals (CIs). The analysis included a univariate model and three progressively adjusted multivariate models. The modeling approach was sequentially structured on the basis of clinical logic and designed to independently evaluate metabolic and immune variables and demonstrate that they do not exhibit collinearity. Model 1 was adjusted for demographic factors, including age, sex, socioeconomic status, and smoking status, Model 2 accounted for metabolic comorbidities, including obesity rate (body mass index ≥ 30), hypertension, dyslipidemia, and ischemic heart disease (IHD), in addition to Model 1, and Model 3 included adjustments for immune-mediated conditions compared to Model 1. Variables representing comorbid conditions that developed after the diagnosis of HS were excluded from the analysis. A patient was defined as having a comorbid disease or medical condition only if the relevant CHS registry data were complete and available. A previous study using the CHS registry showed that body mass index and smoking history were missing for <2% of participants [21].

Statistical analyses were performed using R software (version 4.3.2). All tests were two-sided, with a significance threshold of 0.05. Results were reported with 95% confidence intervals.

## 3. Results

### 3.1. Characteristics of the Study Population

The study population consisted of 10,919 patients with HS and 53,314 age-, sex-, and primary care clinic-matched control subjects without HS (Table 1). The HS group included 4486 males (41.1%) and 6433 females of mean (SD) age 32.2 (13.6) years at diagnosis. Compared to controls, the HS group had a significantly higher proportion of smoking (51.5% vs. 31.4%), obesity (31.3% vs. 14.8%), hyperlipidemia (18.6% vs. 14.5%), hypertension (7.3% vs. 5.5%), and IHD (2.1% vs. 1.6%; all *p* < 0.001). The HS group also had a significantly higher proportion of hypothyroidism (4.8% vs. 3.8%, *p* < 0.001). There was no between-group difference in the Charlson Comorbidity Index (CCI) score (*p* = 0.29).

### 3.2. Characteristics of HS and Control Subjects with T1D

**Figure 1:**
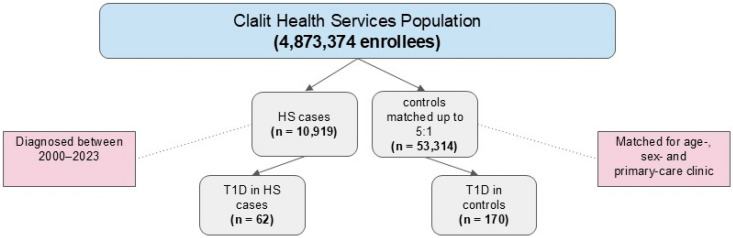
Flow diagram of participant selection in the nested case-control design.

T1D was diagnosed in 62 patients with HS and 170 control subjects (Figure 1 and Table 2). There was no significant difference in the mean age and proportion of males between these subgroups. Patients with HS/T1D comorbidity were more likely than control patients with T1D alone to be smokers (54.8% vs. 35.3%, *p* = 0.01) and had higher proportions of obesity (41.9 vs. 22.9, *p* = 0.008), hypertension (37.1% vs. 17.7%, *p* = 0.003), and IHD (19.4% vs. 7.1%, *p* = 0.01). The two groups had similar proportions of hypo- and hyperthyroidism, celiac disease, and HbA1c levels.

The CCI score of patients with HS/T1D comorbidity was higher than in patients with T1D alone (3.5 (3.1) vs. 2.2 (1.9), *p* = 0.004).

### 3.3. Odds of Developing HS with a Preexisting Diagnosis of T1D

T1D was significantly associated with a diagnosis of new-onset HS (OR 1.80, 95% CI (1.30–2.40), *p* < 0.001). Preexisting T1D was identified in 0.6% of the HS group compared to 0.3% of the control group (*p* < 0.001; Table 3). In all patients with HS/T1D comorbidity, the diagnosis of T1D preceded the diagnosis of HS by a mean (SD) of 13.3 (10.0) years.

Stratification of the patients and controls with T1D showed that in the 20–40-year age group, the presence of T1D was a significant predictor of HS (*p* = 0.01). The odds were even higher in patients older than 40 years (*p* < 0.001), regardless of gender, body mass index, or smoking status (Table 3). In the multivariate logistic regression analysis (Model 1), T1D was associated with increased odds of HS (aOR 1.84, 95% CI (1.40–2.50), *p* < 0.001). After adjustments for metabolic comorbidities (Model 2) and for immune-mediated comorbidities (Model 3), the independent odds of developing HS in patients with T1D remained statistically significant (aOR 1.70, 95% CI (1.50–2.30), *p* < 0.001, and aOR 1.78, 95% CI (1.32–2.41), *p* < 0.001, respectively).

### 3.4. Characteristics of Patients with HS/T1D Comorbidity Compared to HS-Only

The demographic and clinical characteristics, including age, sex, ethnicity, smoking status, and obesity rate, were similar in patients with HS/T1D comorbidity and patients with HS alone (Table 4). Patients with HS/T1D comorbidity had a significantly higher mean CCI score (3.5 (3.1) vs. 0.9 (1.7), *p* < 0.001). Hypertension, hyperlipidemia, and IHD were significantly more prevalent in the HS/T1D comorbidity group (***p*** < 0.001), as were hypothyroidism and celiac disease (***p*** < 0.001).

## 4. Discussion

The present study showed that T1D is associated with new-onset HS regardless of metabolic and autoimmune comorbidities. Compared to patients with HS alone, patients with HS/T1D comorbidity exhibited worse metabolic parameters, including higher proportions of hyperlipidemia, hypertension, obesity, and IHD. They also had an increased proportion of immune-mediated conditions (e.g., hypothyroidism and celiac disease). Compared to patients with T1D alone, patients with HS/T1D were older and had higher rates of hypertension and IHD. In addition, the CCI score was higher in patients with HS/T1D than in patients with either HS or T1D alone.

This is the first population-based study to establish a temporal relationship between T1D and HS and to directly assess the odds of incident HS developing in patients with T1D. Two previous population-based studies proposed an association between T1D and HS [19,20]. The first, a cross-sectional study from Korea, did not evaluate the temporal relationship, nor did it account for clinical and metabolic confounders that could impact the association. In Asian populations, HS has a lower prevalence (0.06%) and a reversed female-to-male ratio (1:1.6) compared to Western populations, and it affects different anatomical regions, limiting the generalizability of the findings [19].

The second study, conducted in Denmark, indicated that T1D tended to be diagnosed before the appearance of cutaneous abscesses, which often served as an interim diagnosis before the eventual identification of HS. The study suggested that patients newly diagnosed with HS may have a higher prevalence of T1D, but it did not establish a direct link between the two conditions, probably owing to the often-extended diagnostic delay in HS [20].

Both population-based studies of the potential link between HS and T1D used unvalidated ICD-10 codes [19,20]. By contrast, we used algorithms developed at the Chronic Disease Center of CHS specifically for T1D diagnosis, integrating ICD codes, prescriptions, and clinical indicators (HbA1c), along with demographic factors, such as age. This comprehensive approach improved the accuracy of diagnosis of T1D and reduced its misclassification as T2D. Additionally, a subset of all T1D diagnoses was manually validated by endocrinologists specializing in diabetes, in which accuracy exceeded 90%. Notably, three out of four diagnostic criteria achieved a 95% accuracy rate, demonstrating the algorithm’s enhanced precision over reliance solely on ICD coding.

The group of patients with HS/T1D comorbidity exhibited a high proportion of metabolic disturbances compared to the broader HS group (Table 2), reflecting a phenotypic profile resembling T2D [22]. Patients with HS/T1D comorbidity had a higher proportion of autoimmune conditions, such as hypothyroidism and celiac disease, than patients with HS alone (Table 4). These findings reflect the autoimmune characteristics commonly associated with T1D [23].

A previous population-based study in the USA found that for patients with HS, each one-unit increase in CCI score was associated with a 25% higher risk of death (OR, 1.25; 95% CI (1.21–1.29)) [24]. In our study, patients with HS/T1D comorbidity had a CCI score that was 2.6 units higher than those with HS alone (3.5 vs. 0.9, ***p*** < 0.001) and 1.3 units higher than those with T1D alone (3.5 vs. 2.2, ***p*** = 0.004). The great comorbidity burden displayed by this subgroup might indirectly suggest a heightened mortality risk.

No theories have so far been advanced to explain the potential association between T1D and HS. Common pathogenic mechanisms of dysregulated inflammatory pathways underlie both diseases. The pivotal pro-inflammatory cytokines in HS, namely, TNF-α and IL-17 [25], are thought to contribute to the onset and progression of T1D by promoting the differentiation and function of autoreactive immune cells that mediate the destruction of pancreatic beta cells [26,27]. Alternatively, an imbalance in the T-helper 17 (Th17)/T-regulatory (Treg) axis is common to both diseases, leading to excessive production of IL-17 and perpetuation of chronic inflammation [28,29]. However, medications that target inflammatory cytokines, which are the mainstay of treatment of HS [30], either have not been tested in T1D or did not show a beneficial effect in postponing the disease in humans despite promising results in mice [31].

In the early stages of T1D, pro-inflammatory signaling is initiated within the endocrine pancreas through the activation of nuclear factor kappa-light-chain-enhancer of activated B cells (NF-κB). NF-κB is considered crucial to the survival and function of β cells, and as such, influences disease progression [32]. Similarly, NF-κB plays a key role in the pathogenesis of HS as a downstream effector of TNF-α, making it a critical target for treatments, particularly in mild to moderate disease [33]. Glucagon-like peptide 1 (GLP-1) receptor agonists are well-documented inhibitors of NF-κB signaling. Recent evidence has increasingly shown them to be effective in managing both HS and T1D. Their therapeutic impact is attributed to dual actions: modulating metabolic processes and exerting significant immunomodulatory effects, addressing key aspects of these two conditions [32,33,34,35,36]. Semaglutide, which is approved for managing T2D and obesity, works by enhancing insulin secretion, thereby reducing glucagon release, lowering blood glucose levels, and suppressing appetite, leading to weight loss [34]. GLP-1 receptor agonists were also found to promote the proliferation of β cells and inhibit their apoptosis in a mouse model [37]. A recent pilot case series published in the *New England Journal of Medicine* reported that semaglutide-treated patients with newly diagnosed T1D experienced improved glycemic control over the course of a year [35]. Additionally, according to a recently published systematic review, the administration of GLP-1 receptor agonists, specifically liraglutide and semaglutide, to patients with HS led to a significant reduction in systemic inflammation that was attributed to the suppression of key inflammatory pathways involving TNF-α, IL-17, and NF-κB. Clinically, the effect of treatment was manifested by a decrease in lesion severity and improvement in quality of life [33,36].

The present population-based study has several limitations. The longitudinal design allowed for assessment of temporality, but its retrospective nature precluded the determination of causation between T1D and subsequent HS. Data on HS severity were unavailable, as the study relied on computerized datasets that did not include this information. As is common in observational studies, unmeasured confounders may introduce bias or residual confounding, potentially affecting the validity of the findings. Additionally, selection and misclassification biases may arise because of reliance on registry data, which depends on the accuracy and completeness of diagnostic coding. To mitigate these limitations, we matched cases and controls on the basis of their primary care clinic to account for healthcare access and shared environmental factors. Although we implemented random manual validation of T1D diagnoses to verify their accuracy and reduce potential misclassification bias, there remained a possibility of residual bias, as not all files could be manually reviewed owing to constraints related to information security and patient privacy. Additionally, it would have been useful to examine HbA1c levels prior to the diagnosis of HS to determine whether the onset of HS coincides with suboptimal diabetes control. The study participants were enrollees in CHS, which covers roughly half the Israeli population. However, the generalizability of these findings to different populations and ethnicities requires further investigation. Future prospective studies could help address these gaps.

## 5. Conclusions

The present large-scale study showed that T1D is associated with new-onset HS. Patients with HS/T1D comorbidity had higher proportions of metabolic conditions (hypertension, obesity, and IHD) and an increased comorbidity burden (as reflected in the CCI score) compared to patients with T1D alone. They also had higher proportions of immune-mediated disorders (celiac disease and hypothyroidism) compared to patients with HS or T1D alone. Dermatologists and endocrinologists should be aware of the potential link between these diseases. Further longitudinal research is necessary to identify metabolic disturbances, such as obesity, and immune-related comorbidities, such as thyroid disorders, that predispose individuals to HS/T1D comorbidity. Additionally, identifying biomarkers, such as HbA1c and C-reactive protein, which may indicate susceptibility to HS in patients with T1D, will facilitate the development of surveillance and therapeutic strategies targeting shared pathways, including those affected by TNF-α inhibitors [38] and GLP-1 receptor agonists.

## Figures and Tables

**Table 1:** Descriptive characteristics of the study population.

Characteristics	HS Group (*n* = 10,919)	Control Group (*n* = 53,314)	*p*-Value
Age at diagnosis (years), mean (SD)	32.2 (13.6)	32.2 (13.7)	0.74
Sex, N (%)			
Male	4486 (41.1)	21,966 (41.2)	0.83
Female	6433 (58.9)	31,348 (58.8)	
SES, mean score (SD)	5.4 (2.3)	5.4 (2.3)	0.28
Obesity, *n* (%) ^1^	3414 (31.3)	7889 (14.8)	<0.001
Smoking, *n* (%)	5627 (51.5)	16,724 (31.4)	<0.001
CCI score, mean (SD)	0.9 (1.7)	1.0 (1.7)	0.29
Hyperlipidemia, *n* (%)	2032 (18.6)	7735 (14.5)	<0.001
Hypertension, *n* (%)	791 (7.3)	2923 (5.5)	<0.001
Hyperthyroidism, *n* (%)	70 (0.5)	268 (0.6)	0.07
Hypothyroidism, *n* (%)	522 (4.8)	2047 (3.8)	<0.001
Celiac disease, *n* (%)	43 (0.4)	221 (0.4)	0.9
IHD, *n* (%)	225 (2.1)	852 (1.6)	<0.001

^1^ Body mass index ≥ 30. CCI, Charlson Comorbidity Index; HS, hidradenitis suppurativa; IHD, ischemic heart disease; SD, standard deviation; SES, socioeconomic status.

**Table 2:** Characteristics of patients with T1D in the HS and control groups.

Characteristics	Patients with HS and T1D (*n* = 62)	Control Subjects with T1D (*n* = 170)	*p*-Value
Age at T1D diagnosis (years), mean (SD)	21.2 (15.5)	19.8 (11.8)	0.50
Sex, *n* (%)			
Male	27 (43.5)	71 (41.8)	0.90
Female	35 (56.5)	99 (58.2)	
Obesity, *n* (%)	26 (41.9)	39 (22.9)	0.008
Smoking, *n* (%)	34 (54.8)	60 (35.3)	0.01
CCI score, mean (SD)	3.5 (3.1)	2.2 (1.9)	0.004
Celiac disease, *n* (%)	5 (8.1)	11 (6.5)	0.90
Hyperthyroidism, *n* (%)	0 (0)	5 (2.9)	0.40
Hypothyroidism, *n* (%)	18 (29)	35 (20.6)	0.20
Hyperlipidemia, *n* (%)	39 (62.9)	102 (60)	0.80
Hypertension, *n* (%)	23 (37.1)	30 (17.7)	0.003
IHD, *n* (%)	12 (19.4)	12 (7.1)	0.01

CCI, Charlson Comorbidity Index; HS, hidradenitis suppurativa; IHD, ischemic heart disease; SD, standard deviation; SES, socioeconomic status; T1D, type 1 diabetes.

**Table 3:** Subgroup analysis of study participants with T1D.

Subgroups	Patients with HS and T1D *n* (%)	Control Subjects with T1D *n* (%)	OR, Unadjusted (95% CI)	*p*-Value, Unadjusted
All	62 (0.6)	170 (0.3)	1.80 (1.30–2.40)	<0.001
Sex				
Male	27 (0.6)	71 (0.3)	1.88 (1.21–2.90)	0.005
Female	35 (0.5)	99 (0.3)	1.74 (1.18–2.60)	
Age (years)				
<20	6 (0.4)	21 (0.3)	1.40 (0.56–3.50)	0.47
20–40	37 (0.6)	114 (0.4)	1.60 (1.10–2.30)	0.01
≥40	19 (0.7)	35 (0.3)	2.60 (1.49–4.53)	<0.001
Smoking status				
Smoker	34 (0.6)	60 (0.3)	1.87 (1.17–3.00)	0.009
Non-smoker	28 (0.6)	110 (0.3)	2.10 (1.27–3.49)	
Obesity *				
Yes	26 (0.8)	39 (0.5)	1.77 (1.34–2.35)	<0.001
No	36 (0.5)	131 (0.3)	1.96 (1.34–2.89)	

* Body mass index ≥ 30. CI, confidence interval; HS, hidradenitis suppurativa; OR, odds ratio; T1D, type 1 diabetes.

**Table 4:** Characteristics of patients with HS/T1D comorbidity as compared to patients with HS alone.

Characteristics	Patients with HS and T1D (*n* = 62)	Patients with HS Only (*n* = 10,857)	*p*-Value
Age at diagnosis of HS (years), mean (SD)	35.1 (17.1)	32.2 (13.6)	0.10
Sex			0.8
Male	27 (43.5)	4459 (41.1)	
Female	35 (56.5)	6398 (58.9)	
SES, mean score (SD)	5.2 (2.5)	5.4 (2.3)	0.50
Obesity, *n* (%)	26 (41.9)	3388 (31.2)	0.09
Smoking, *n* (%)	34 (54.8)	5593 (51.5)	0.70
CCI score, mean (SD)	3.5 (3.1)	0.9 (1.7)	<0.001
Hyperlipidemia, *n* (%)	39 (62.9)	1993 (18.4)	<0.001
Hypertension, *n* (%)	23 (37.1)	768 (7.1)	<0.001
IHD, *n* (%)	12 (19.4)	213 (2.0)	<0.001
Hyperthyroidism, *n* (%)	0 (0)	70 (0.6)	1.00
Hypothyroidism, *n* (%)	18 (29.0)	504 (4.6)	<0.001
Celiac disease, *n* (%)	5 (8.1)	38 (0.4)	<0.001

CCI, Charlson Comorbidity Index; HS, hidradenitis suppurativa; IHD, ischemic heart disease; SD, standard deviation; SES, socioeconomic status; T1D, type 1 diabetes.

## Data Availability

The data supporting this study are available upon reasonable request from the corresponding author. Restrictions apply to the availability of these data due to patient privacy.

## Abbreviations

The following abbreviations are used in this manuscript:

CHS	Clalit Health Services
GAD	Glutamic acid decarboxylase
HS	Hidradenitis suppurativa
IHD	Ischemic heart disease
IL-1ꞵ	Interleukin-1 beta
OR	Odds ratio
TNF-α	Tumor necrosis factor alpha
T1D	Type 1 diabetes
T2D	Type 2 diabetes
